# Personality traits or emotional dysregulation: a multiple mediation analyses of adolescent depression

**DOI:** 10.1186/s40479-025-00302-6

**Published:** 2025-08-07

**Authors:** Tore Aune, Leo Wolmer, Sigrid Flatås Aune, Daniel Hamiel, Hans M. Nordahl

**Affiliations:** 1 Faculty of Nursing and Health Sciences, Nord Universitetet, Levanger, Norway; 2 Norwegian Directorate of Children, Youth, and Family Affairs, Steinkjer, Norway; 3 Baruch Ivcher School of Psychology, Reichman University, Herzlyia, Israel; 4 Norwegian University of Science and Technology, Trondheim, NO-7491 Norway; 5 Donald J. Cohen & Erving B. Hariss Resilience Center, Tel-Aviv, Israel; 6 Department of Mental Health, NTNU, Trondheim, Norway; 7 Department of Acute Psychiatry, St. Olavs Hospital HF, Trondheim University Hospital, Trondheim, Norway

**Keywords:** Personality traits, Stressful life events, Depression, Mediation, Adolescents, Longitudinal

## Abstract

**Background:**

In a similar way to depression, the development of borderline personality traits seems closely linked to the experience of Stressful Life Events (SLEs). This study examined the extent to which emotion regulation and personality traits simultaneously mediate and significantly attenuate the direct path between SLEs and depressive symptoms.

**Method:**

Parallel and serial multiple mediation models were employed to analyze a sample of 196 adolescents assessed twice over a 12-month period. A regression-based path analysis approach was employed to simultaneously estimate the specific indirect effects of emotion regulation and borderline traits on the direct relationship between SLEs and depression.

**Results:**

In a parallel multiple mediation model, both borderline traits and emotion regulation fully mediated the direct relationship between SLEs and depressive symptoms. The serial multiple mediation model also showed complete mediation. In this model, the indirect pathway from SLEs to depressive symptoms, first through borderline traits and subsequently through emotion regulation, accounted for 70.8% of the total indirect effect. In contrast, the indirect effects via borderline traits and emotion regulation accounted for 24% and 5.3% of the total indirect effects, respectively.

**Conclusion:**

To strengthen clinical practice, early identification of borderline personality traits combined with targeted interventions to address specific symptoms may enhance emotional regulation and reduce the risk of depressive symptoms in vulnerable adolescents.

## Background

Depression in adolescents is a significant public health concern [1] owing to its high prevalence rate, early onset, and impact on youth, families, and communities [2]. Increases in the prevalence of depressive symptoms among young people have been linked to the stress of the COVID-19 pandemic (36% of those aged 18–29) [3]. Daly [4] also reported a significant increase in the prevalence of depression among adolescents, from 8.1% in 2009 to 15.8% in 2019, in line with reports of increasing stress, anxiety, self-harm, and suicidality [5, 6]. Adolescents often experience stressful and potentially traumatic events, which significantly increase their risk of developing psychological disorders [7]. A study by Frazier et al. [8] found that 85% of U.S. undergraduate students had experienced at least one stressful life event (SLE) in their lifetime. Moya-Higueras et al. [9] assessed a sample of Spanish adolescents and demonstrated that major dependent noninterpersonal SLEs were the best predictors of externalizing psychopathology. In contrast, major independent noninterpersonal SLEs were the best predictors of internalizing symptoms and low life satisfaction. Researchers have studied the link between these events and psychopathology by examining the effects of controllable and uncontrollable life events. A meta-analysis examining the relationship between SLEs and internalizing and externalizing psychopathology found that the controllability of the events did not affect the results [10]. However, the study showed that experiencing multiple SLEs events during adolescence is a risk factor for developing internalizing and externalizing symptoms, thereby increasing the likelihood of specific psychiatric disorders. Reports have shown the experience of SLEs preceding the onset of depressive symptoms [11–13] or subsequent reoccurrences [14, 15]. This association was observed in both cross-sectional and longitudinal studies. Interestingly, the authors did not observe significant differences in the strength of these associations based on the assessment method, whether through interviews or checklists [10]. Individuals with depression are estimated to be between 2.5 and 9.4 times more likely than the average to have experienced an SLE prior to the first onset of depression, making recent stressor exposure one of the most decisive risk factors for depression in community samples [15, 16]. Although these findings are consistent, there is some debate regarding the strength of the relationship between the SLEs and depressive symptoms. More precisely, how this association is mediated and moderated by other variables, such as emotion regulation, specifically among adolescents, remains unclear. Therefore, the primary focus of current research is to examine mediating factors that contribute to the link between SLEs and depressive symptoms in adolescents.

Borderline personality disorder (BPD) is characterized by a persistent pattern of emotional instability, impulsivity, unstable self-image, and interpersonal impairment [14], and it has a comorbidity with depressive symptoms in adolescence [17]. BPD is a reliable and valid construct among adolescents [18, 19], and its prevalence is comparable to that found among adults [20]. These symptoms peak around the ages of 14–17 years, a critical risk period for intervening and modifying the trajectory of the disorder [21]. Like depression, the development of borderline traits seems closely linked to the experience of SLEs, neglect, and inconsistent parentship [22]. Traumatic experiences appear to induce difficulties in emotional regulation and interfere with emotional development [23].

Impaired emotion regulation is a hallmark feature of major depressive episodes (APA, 2013) and the core feature of BPD. Emotion regulation mediates the relationship between stress/trauma and depression in adolescents [24, 25], undergraduates [26], adult patients [27] and non-patients [28]. McLaughlin, Hatzenbuehler, and Hilt [29] showed that emotion dysregulation mediates the relationship between SLEs and both anxiety and depressive symptoms in older children (sixth to eighth grade). However, they examined the indirect effect (IE) using Sobel’s test, which has been criticized for mediation analyses [30, 31]. Instead, a bootstrap method, as used by Stikkelbroek et al. [26] and Aune et al. [25], has been recommended [30]. Nevertheless, these researchers used only one mediator in the model with a cross-sectional design. Therefore, no temporal conclusions or causal inferences could be drawn. Schäfer et al. [32] noted a lack of studies exploring this mediating relationship with a nonclinical sample of older adolescents, especially with longitudinal designs [25].

Several compelling arguments exist for using borderline traits and emotion dysregulation as mediators in the same model. Theoretically and conceptually, these constructs are closely interrelated [33]. According to the biosocial theory of borderline personality disorder (BPD), emotion dysregulation not only arises from but also maintains borderline personality features (BPF) [34]. These theoretical links provide a strong rationale for examining these constructions as mediators. There is also empirical evidence demonstrating significant associations between BPD symptoms, emotion dysregulation, and various outcomes [35, 36]. Herr et al. [36] found that difficulties with emotion regulation fully mediated the relationship between BPD symptom severity and interpersonal problems. Scott et al. [35] demonstrated that difficulties with emotion regulation fully mediated associations between BPD symptoms at baseline and later psychological and physical aggression. Additionally, BPD symptoms predict more significant emotion dysregulation [37].

While studies often examine BPD traits and emotion dysregulation as separate mediators, limited research directly compares their effectiveness when considered together. By including both BPD traits and emotion dysregulation as mediators in the same model, we can assess their unique and combined effects. BPD traits encompass aspects of the disorder that extend beyond emotion dysregulation, including emotion sensitivity, affective intensity, affective lability, emotional vulnerability, identity disturbance, and, importantly, fear of abandonment [38].

We will introduce two models to examine the mediation effects of BPD traits and emotion regulation on the direct path between SLEs and depressive symptoms. A parallel mediation model allows for the possibility that BPD traits and emotion dysregulation each independently mediate between SLEs and depressive symptoms. This approach is supported by evidence that, while closely related, BPD traits and emotion dysregulation can have unique and additive effects on psychological outcomes [38].

Applying a serial multiple mediation model is theoretically justified by the biosocial model of BDP [34], which posits that BPD traits can lead to increased emotion dysregulation, which in turn contributes to depressive symptoms. BPD features may precede and exacerbate emotion regulation difficulties, supporting a potential causal chain from SLEs → borderline traits → emotion dysregulation → depression [38]. In a serial mediation model, one could argue that BPD traits may act as a distal factor that affects emotion regulation. Specifically, individuals with high levels of BPD traits may have more negative appraisals of their emotions, rely more on avoidance or impulsive strategies to regulate their emotions, and experience more conflict or rejection in their relationships, which can further exacerbate their emotional dysregulation.

Given the theoretical and empirical links between BPD traits and emotion dysregulation and the possibility of their independent effects, it was deemed essential to test both models.

Findings from such analyses will enhance our understanding of the relative contributions of BPD traits and emotion dysregulation, which can inform treatment approaches. For example, dialectical behavior therapy (DBT) targets both BPD symptoms broadly and emotion regulation skills precisely [34]. In conclusion, simultaneously examining BPD traits and emotion dysregulation as mediators in the direct path from SLE to depressive symptoms provides a more comprehensive understanding of the mechanisms underlying BPD symptoms and emotion dysregulation and their associated outcomes.

To overcome some methodological limitations of previous studies, this study examined the specific effects of two intermediate variables, emotion regulation, and borderline traits, on the direct path from SLEs to depression. It employs a longitudinal design, utilizing parallel and serial multiple mediator models. Also, applying a serial multiple mediation model has some advantages. It provides a more comprehensive understanding of the underlying mechanisms through which the independent variable influences the dependent variable. Additionally, the serial multiple mediation model enables the investigation of indirect and direct effects at each stage of the mediational process, providing a more nuanced analysis of the relationships involved.

We examined the following hypotheses. First, we anticipate a statistically significant direct effect between SLEs and depressive symptoms. Second, using a parallel multiple mediator model, we expect to find a moderate bivariate correlation between the two mediators: emotion regulation and BPD traits. Third, in a serial multiple mediator model, we expect that BPD traits, through emotion dysregulation, will mediate the relationship between SLEs and depressive symptoms.

## Methods

### Procedure

The Vestfold County (Norway) school authorities supported this study, and information about the survey was made available on the county’s official website. Representatives from the school authorities informed the students about the survey during class, and written information and consent forms were distributed. Following Norwegian ethical requirements, students aged 16 years and older provided informed consent on their behalf. At the same time, those under 16 were permitted to participate with consent forms signed by themselves and their parents. Participation was voluntary, and students who did not return the consent form within one week received one reminder.

The participants were assessed twice, using the same assessment battery, with a 12-month interval between the first assessment (Assessment I) and the second assessment (Assessment II). In the parallel multiple mediation model, stressful life events (the predictor) and two mediators—borderline traits and emotion regulation—were evaluated during Assessment Point (I) Depressive symptoms (outcome variable) were measured one year later at Assessment Point (II) In the serial multiple mediation model, stressful life events and borderline traits were assessed at Assessment Point I. In contrast, emotion regulation and stressful life events were evaluated at Assessment Point II.

### Participants

A sample of first- and second-year high school students was recruited from three senior high schools in Vestfold County, Norway. Approximately 25% of the participants reported that they were not born in Norway, while 18% indicated that they do not speak Norwegian at home most days (see Table 1). Out of 235 eligible students, 196 (83.4%) participated in two assessment points. The mean age of the participants was 16.9 years (SD = 1.84), with 65.8% (*n* = 129) identifying as female.

**Table 1:** Demographic characteristics of participants.

Characteristic	Mean	SD	*n*	%
Age	16.87	1.76		
Gender				
Female			129	65.8
Male			67	34.2
School grade				
VG1 (first year at high school/11th year at school)			126	64.3
VG2 (second year at high school/12th year at school)			66	33.7
Not reported			4	2.0
Living with adults				
With mother and father, who live together			103	52.6
With mother and father, who do not live together (50/50)			15	7.7
Mostly with mother			36	18.4
Mostly with father			5	2.6
With mother and her new partner			15	7.7
With father and his new partner			3	1.5
With partner or friend			3	1.5
With other adults			6	3.1
Living alone			6	3.1
In institution			3	1.5
Not reported			1	0.5
Place of birth				
Born in Norway			146	74.5
Not born in Norway			48	24.5
Not reported			2	1.0
Language use				
Speaks Norwegian at home all or most days			153	78.1
Does not speak Norwegian at home at all or most days			36	18.4
Not reported			7	3.6
Family’s economic situation during the last two years.				
Good all the time			72	36.7
Good most of the time			60	30.6
Neither good nor bad			36	18.4
Bad most of the time			17	8.7
Bad all the time			3	1.5
Not reported			8	4.1

### Measures

The *Difficulties in Emotion Regulation Scale* (DERS) [39] is a 36-item self-report measure designed to assess difficulties in emotion regulation. Each item is rated on a scale from 1 (rarely [0–10%]) to 5 (almost always [91–100%]), with higher scores indicating more incredible difficulty in emotion regulation. Exploratory factor analysis has suggested six- and seven-factor structures for DERS [39]. The six-factor model has been found to adequately fit various populations, including adolescents [40].

Research has demonstrated significant positive associations between DERS scores and the symptoms of various psychological disorders [41, 42]. Gratz and Roemer [39] have validated the DERS against other mood regulation scales, such as the Negative Mood Regulation Scale, showing strong construct validity (*r* = − 0.34 to − 0.69, all *p* < 0.01). Due to constraints on the total number of items that could be included in the survey booklet administered to participants, we were required to reduce the length of the DERS. As a result, two factors (impulse control difficulties and lack of emotional clarity) were excluded from our assessment. Thus, in the present study, four factors of the original DERS were assessed and used to calculate the total DERS score: (1) Nonacceptance of emotional responses (e.g., “When I’m upset, I become angry with myself for feeling that way”); (2) Difficulty engaging in goal-directed behavior (e.g., “When I’m upset, I have difficulty getting work done”); (3) Lack of emotional awareness (e.g., “I pay attention to how I feel”); and (4) Limited access to emotion regulation strategies (e.g., “When I’m upset, I believe that I’ll end up feeling very depressed”). Cronbach’s alpha for the DERS (25 items) was 0.87.

The *Depression Anxiety Stress Scale* (DASS-21) is a 21-item self-report measure that assesses three facets of negative emotion: depression, anxiety, and stress/tension [43]. Each subscale contains seven items. In the current study, we used the DASS-21 depression subscale (7 items), which demonstrated a very high degree of internal consistency in our sample (Cronbach’s α = 0.90). Respondents recorded the presence of a symptom over the previous week on a scale from 0 (did not apply to me) to 3 (applied to me very much or most of the time). Because DASS-21 is a short form of DASS-42, the final score of each subscale is multiplied by two (total scores range from 0 to 42). A cut-off score of 14 has been recommended to categorize clinical moderate or higher depressive symptoms or depression [44].

The *Borderline Personality Feature Scale for Children* (BPFSC11) is an 11-item inventory that assesses borderline traits among children and adolescents [45]. Items are rated on a Likert-type scale of 1 (*never true*) to 5 (*always true*) (total score 11–55) and capture behaviors reflective of core BPD features (e.g., affective instability, identity problems, and negative relationships) [45]. The BPFS-C-11 has shown good criterion validity of test score interpretations, internal consistency, test-retest reliability, and satisfactory Cronbach’s of 0.79-0.85 [45, 46]. Fossati et al. [45] reported a six-month test-retest correlation of 0.50 in a sample of 817 adolescents. Furthermore, a significant correlation (*r* = 0.64, *p* < 0.001) between the BPFSC11 and a clinical interview indicated adequate convergent validity [47]. Sharp et al. [45] demonstrated partial invariance of the BPFSC11 across genders and full longitudinal invariance from late adolescence to young adulthood in a community sample. In this study, Cronbach’s alpha for the BPFSC11 (11 items) was 0.87.

*The Risk Factor Questionnaire (RFQ)* evaluates 27 stressful life events (SLEs) that have occurred in the past 12 months [48]. Each event is recorded as either a simple yes or no (e.g., serious illness, the serious illness of close family members, severe physical abuse, severe verbal violence, threats, or discrimination). The total RFQ score reflects the number of SLEs experienced, with a possible range from 0 to 27. The RFQ has been validated in several studies, demonstrating that SLEs are associated with PTSD and anxiety. Furthermore, it shows that accumulating risk factors increase vulnerability when facing future stressful or traumatic events [48, 49].

### Statistical analysis

Statistical analyses were conducted using SPSS Statistics for Windows (v. 27.0; IBM SPSS, Armonk, NY, USA). Means and standard deviations for the predictor variable RFQ score, the outcome variable DASS (depressive symptoms), and the two mediators DERS (emotion regulation) and BPFSC-11 (borderline traits) scores are reported. Correlations between the mediators and the outcome variable at two assessment points are also reported. Hayes’ [30] PROCESS macro for SPSS (v. 3.5; SPSS Inc, Chicago, IL, USA) was used to assess mediation. Hayes’ PROCESS macro employs a regression-based path analysis approach. To test for statistical significance and obtain the 95% bias-corrected confidence level for the indirect effect (IE), maximum likelihood bootstrapping was performed by estimating 5,000 bootstrap samples for the hypothesized model. Bootstrapping, a resampling method, is recommended for estimating mediation [30]. A 95% confidence interval (CI) that does not include 0 indicates a significant IE (*p* < 0.05). All reported regression coefficients are unstandardized.

#### Model 1 (A parallel multiple mediation model)

A parallel multiple mediator model (Hayes’ PROCESS model 4) tested the specific IE of the two mediators, borderline traits and emotion regulation, while controlling for the effect of each other. The cumulative specific IE yields the total effect of X on Y through the mediators. The direct effect of X quantifies the estimated difference in Y for each unit difference in X, independent of the other mediator [30]. A pairwise comparison tested whether the two IEs differed significantly. In this model, the mediators (DERS and DASS) were assessed at assessment point I, while the dependent variable was assessed at assessment point II, one year later.

#### Model 2 (A serial multiple mediation model)

The serial multiple mediation model (PROCESS model 6) tested the direct and indirect effects of SLEs on depressive symptoms, modeling a process where SLEs lead to borderline traits, which in turn lead to emotional dysregulation, eventually resulting in depressive symptoms. As in the parallel model, the total effect of the consequent variable on the antecedent variable is partitioned into direct and indirect components. The direct effect is the estimated difference in depressive symptoms for each unit difference in SLEs, considering equal mediator values. The IEs are derived by multiplying the regression weights for each step on the indirect pathway, indicating the difference in SLEs between the two mediators of depressive symptoms. The total IE is the sum of the specific IEs, while the direct effect and total IE combine to form the model’s total effect. This model comprises three independent effects (IEs) and one direct effect. SLEs and BPFSC-11 were measured at assessment point I, while DERS and DASS scores were measured at assessment point II, 12 months later.

### Ethics

This research adhered to the ethical guidelines of the World Medical Association (Declaration of Helsinki) for experiments involving humans. It was approved by the Norwegian Regional Committee for Medical and Health Research Ethics (REK 2017/1938) and the Norwegian Centre for Research Data (#53960). After providing a complete study description, participants provided written informed consent. All participants were assured of confidentiality.

## Results

An assessment of the standardized regression residuals for the dependent variable, depression, through a normal P-P plot revealed a slight deviation from the normality line. However, the scatterplot displayed no discernible pattern, and the residuals were approximately evenly distributed above and below the vertical and horizontal axes, indicating that the minimum standard for validating the homoscedasticity assumption has been met. In mediation, multicollinearity among predictor variables is commonly expected, although it is acknowledged as a concern specifically in the context of multiple regression [30].

In examining collinearity, the analysis identified DASS (depressive symptoms assessed at assessment point II) as the dependent variable, with RFQ, DERS, and BPFSC-11 as the predictor variables. The results showed tolerance and variance inflation factor (VIF) values as follows: RFQ had a tolerance of 0.834 and a VIF of 1.199; BPFSC11 I showed tolerance of 0.310 and a VIF of 3.222; DERS I had a tolerance of 0.303 and a VIF of 3.305; DERS II had a tolerance of 0.338 and a VIF of 2.961; and BPFSC11 II displayed a tolerance of 0.387 and a VIF of 2.583. The correlations among these variables are presented in Table 2.

**Table 2:** Pearson correlations among the risk factor questionnaire (RFQ), depression anxiety stress scale (DASS), difficulties in emotion regulation scale (DERS), and the borderline personality feature scale for children (BPFSC11) and scale statistics for the various scales

	Mean	SD	Skewness	Kurtosis	RFQ	DASS I	DASS II	DERS I	DERS II	BPFSC I
RFQ	3.16	2.56	1.36	2.78						
DASS I	10.17	10.65	1.05	0.25	0.376					
DASS II	9.37	11.75	1.29	0.65	0.237	0.629				
DERS I	95.34	26.26	0.51	− 0.23	0.339	0.766	0.562			
DERS II	93.11	25.76	0.55	− 0.14	0.259	0.639	0.751	0.754		
BPFSC I	28.72	9.41	0.07	− 0.71	0.386	0.725	0.570	0.757	0.641	
BPFSC II	28.38	9.83	0.04	−0.67	0.316	0.621	0.641	0.603	0.694	0.713

All correlations were statistically significant at the.01 level

RFQ, DASS I, DERS I, and BPFSC I were assessed at assessment point I, whereas DASS II, DERS II and BPFSC II were assessed at assessment point II (12 months later)

### Participant demographics and clinical characteristics

Participant demographics and clinical characteristics are summarized in Table 1.

An independent samples t-test demonstrated no statistically significant differences between those who participated only at assessment point I and those who participated at both assessment points, either on the three clinical scales RFQ *t(233)* = 0.62, *p* > 0.05, DERS I *t(230)* =–0.01, *p* > 0.05, or BPFSC11 *t(61.005)* = 1.32, *p* > 0.05 or on the demographic variable “family economic situation” *t(224)* = 1.62, *p* > 0.05.

The correlation matrix for all variables is presented in Table 2. None of the variables were severely skewed or kurtotic. Emotion regulation (DERS II) was positively correlated with depressive symptoms (DASS) (*r* = 0.75, *p* < 0.001), and borderline traits (BPFSC11 I) was positively associated with emotion regulation (DERS I) (*r* = 0.76, *p* < 0.001). Both mediators were assessed at the assessment point I. Follow-up analyses employing an independent samples t-test showed significant differences across gender for the DERS I (*t*_(192)_ = 3.50, *p* < 0.001), DERS II (*t*_(191)_ = 3.76, *p* < 0.001), BPFSC11 I (*t*_(191)_ = 2.61, *p* < 0.01), BPFSC11 II (*t*_(192)_ = 3.00, *p* < 0.01), and DASS I (*t*_(193)_ = 3.14, *p* < 0.01) and DASS II (*t*_(193)_ = 2.14, *p* = 0.03), but not for the RFQ (*t*_(193)_ =–0.42, *p* = 0.67). The mean and standard deviations for boys and girls were as follows: DERS I, M = 86.47, SD = 24.39/M = 99.95, SD = 26.10; DERS II, M = 83.76, SD = 24.20/M = 97.84, SD = 25.21; BPFSC11 I, M = 26.33, SD = 9.27/M = 30.01, SD = 9.25; BPFSC11 II, M = 25.45, SD = 10.22/M = 29.85, SD = 9.32; DASS I, M = 6.83, SD = 9.06/M = 11.94, SD = 1.02; DASS II, M = 6.98, SD = 10.42/M = 10.65, SD = 12.23; and RFQ, M = 3.31, SD = 2.62/M = 3.11, SD = 2.54, respectively.

### Model 1: the parallel multiple mediation model

**Fig. 1:**
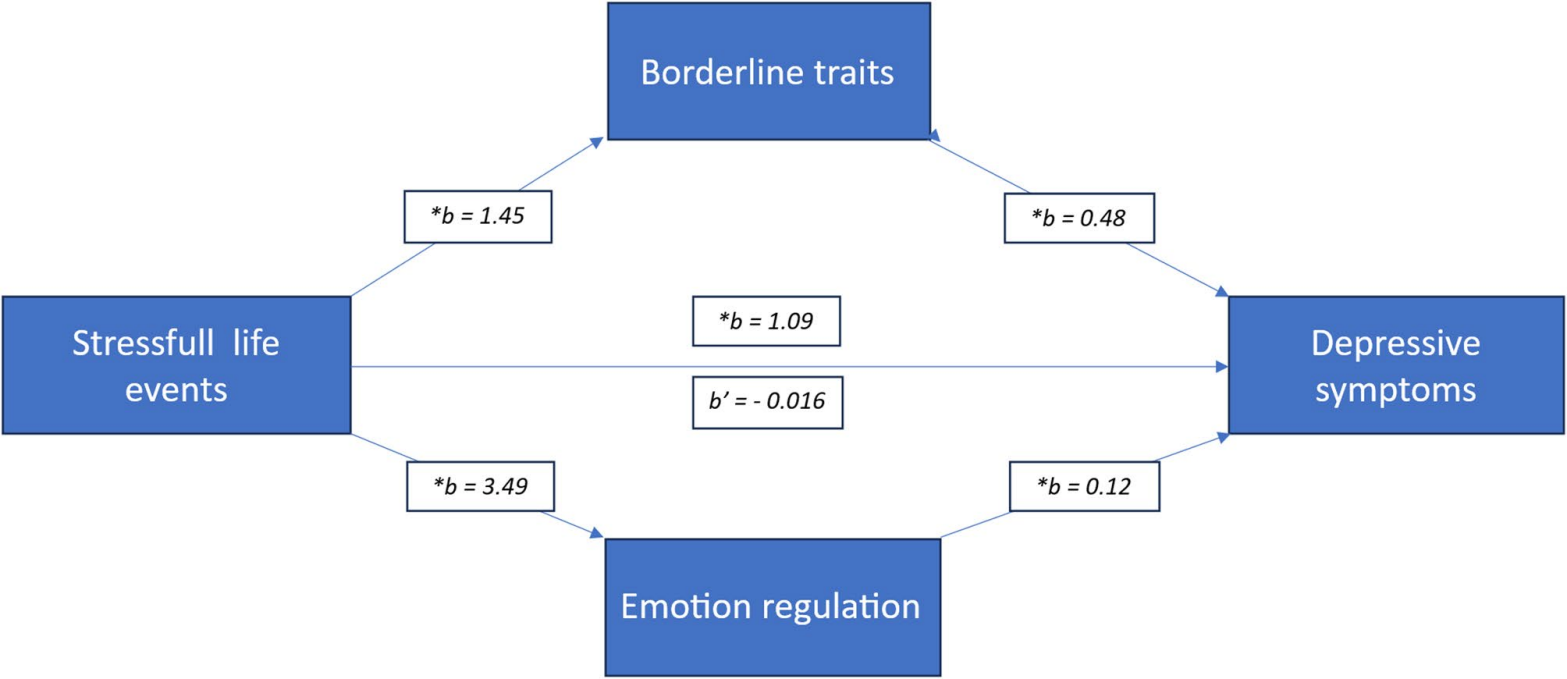
Parallel multiple mediation model with path estimates. Asterisks indicate significant estimates for the various slopes. *b*’ =–0.0016 is the effect of the path from SLEs to depressive symptoms after controlling for the two mediators

Figure 1 shows the parallel multiple mediation model with SLEs as the antecedent variable, depressive symptoms as the consequent variable, and the two intermediate variables borderline traits and emotion regulation

Figure 1 shows the model of the relationship between SLEs and depressive symptoms, mediated by emotion regulation and borderline traits. This model explained 37.9% of the total variance, *F*_4, 187_ = 28.50, *p* < 0.0001. Using sex as a covariate in this model, we found a nonsignificant difference between males and females, *t* (187) =–0.10, *p* = 0.92.

We tested a mediation model using the PROCESS macro, version 3.5. We utilized bootstrap standard errors from 5000 random draws to calculate the standard error of the IE. The outcome variable was specified as depressive symptoms (DASS), the predictor variable was SLEs (RFQ), and the two mediators were emotion regulation (DERS) and borderline traits (BPFSC11). We expected RFQs to affect DASS by reducing DERS and BPFSC11 scores.

The direct path between SLEs and depressive symptoms was statistically significant (*b* = 1.09, *p* < 0.001), indicating that students scoring higher on SLEs were more likely to report more depressive symptoms. There was also a positive and robust relationship between SLEs and emotion regulation (*b* = 3.49, *p* < 0.001) and between emotion regulation and depressive symptoms (*b* = 0.12, *p* < 0.01), indicating that a more significant number of SLEs predicted poorer emotion regulation and this, in turn, predicted increased depressive symptoms.

We also found positive relationships between SLEs and borderline traits (*b* = 1.45, *p* < 0.001) and between borderline traits and depressive symptoms (*b* = 0.48, *p* < 0.001).

When we controlled for the mediation variables DERS and BPFSC11, a reduced and statistically nonsignificant direct effect (*b* =–0.02, *p* = 0.96) was found between SLEs and depressive symptoms, demonstrating a full mediation. Moreover, we observed a statistically significant specific IE (IND = 0.41, BootSE = 0.16, 95% CI 0.12–0.76), demonstrating mediation between SLEs and depressive symptoms via emotion regulation. We also observed a robust and statistically significant specific IE (IND = 0.69, BootSE = 0.19, 95% CI 0.38–1.10), demonstrating mediation between SLEs and depressive symptoms via borderline traits. The total IE was IND = 1.10, BootSE = 0.25, 95% CI 0.67–1.63, whereas borderline traits (BPSC11) and emotion regulation (DERS) accounted for 61% and 39% of the total effect, respectively. However, pairwise comparisons between the two mediators showed a statistically nonsignificant effect difference between the intermediary (DERS and BPFSC11) variables (INED = 0.28, BootSE = 0.25; 95% CI = − 0.18–0.82).

### Model 2: the serial multiple mediation model

**Fig. 2:**
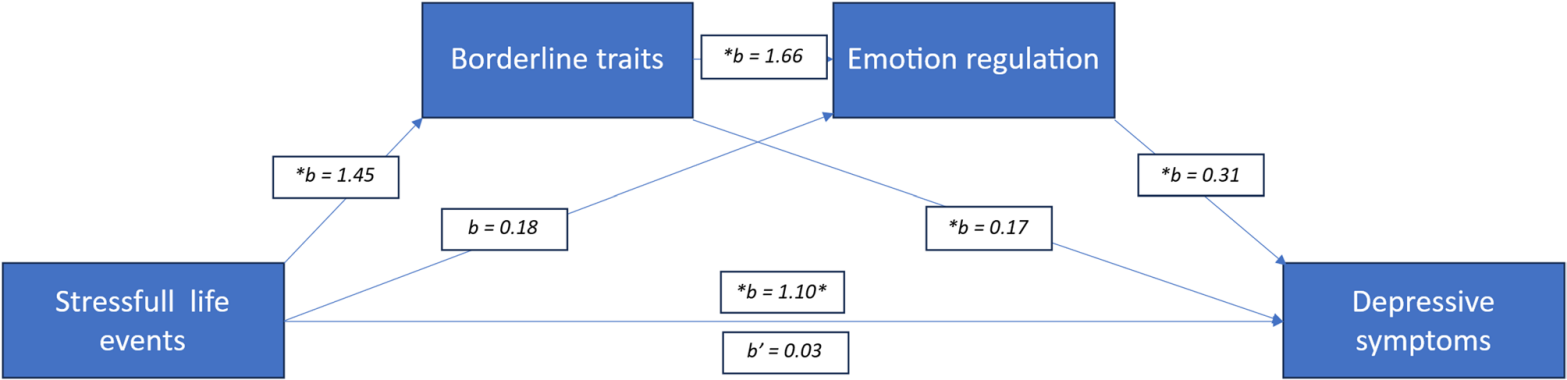
Serial multiple mediation model with path estimates. Asterisks indicate significant estimates for the various slopes. *b*’ = 0.03 is the effect of the path from SLEs to depressive symptoms after controlling for the mediators

Figure 2 shows the serial multiple model of the relationship between SLEs and depressive symptoms, mediated by borderline traits and emotion regulation, where borderline traits were assessed one year before the assessment of emotion regulation. As in model 1, the predictor variable was SLEs, and the outcome variable was depressive symptoms. This model explained 57.7% of the total variance, *F*_4, 185_ = 63.02, *p* < 0.0001. Using sex as a covariate revealed a nonsignificant difference between the sexes, *t* (187) = 1.45, *p* = 0.25.

First, SLEs were positively and statistically significantly associated with borderline traits (*b* = 1.47, *p* < 0.001). By contrast, the path from SLEs to emotion regulation was not statistically significant (*b* = 0.18, *p* = 0.76). However, the path from borderline traits to emotion regulation was robust, positive, and statistically significant (*b* = 1.66, *p* < 0.001), indicating that stronger borderline traits predict more problems with emotion regulation. Moreover, both emotion regulation (*b* = 0.31, *p* < 0.001) and borderline traits (*b* = 0.18, *p* < 0.05) predicted depressive symptoms, demonstrating that both more vital borderline traits and less emotion regulation capacity are associated with an increase in depressive symptoms.

When we controlled for the mediation variables DERS and BPFSC11, a reduced and statistically nonsignificant direct effect (*b* = 0.03, *p* = 0.89) was found between SLEs and depressive symptoms, demonstrating full mediation for this model, too. We observed a statistically significant specific IE (IND = 0.26, BootSE = 0.13, 95% CI 0.02–0.54), demonstrating mediation between SLEs and depressive symptoms via borderline traits. As another result, we observed a statistically nonsignificant specific IE (IND = 0.06, BootSE = 0.18, 95% CI − 0.30–0.41), demonstrating nonsignificant mediation between SLEs and depressive symptoms through emotion regulation. By contrast, a robust, positive, and statistically significant specific IE was revealed whereby SLEs increase borderline traits, which in turn reduce the ability to regulate emotionally, resulting in depressive symptoms as the final consequent *b* = 0.75, BootSE = 0.18, 95% CI 0.44–1.14.

Table 3 shows the mediation estimates for the serial multiple mediation model. The total IE was IND = 1.06, BootSE = 0.28, 95% CI 0.55–1.6, whereas the two paths from SLEs via borderline traits and emotion regulation to depressive symptoms accounted for 24% and 5.3% of the total effect, respectively. The path from SLEs via borderline traits through emotion regulation to depressive symptoms accounted for 70.7% of the total IE. Pairwise comparisons between the two mediators show a statistically nonsignificant difference in effects between the two paths from SLEs to depressive symptoms—via borderline traits and via emotion regulation (IND = 0.20 BootSE = 0.22; 95% CI–0.24–0.65). By contrast, the path to depressive symptoms via borderline traits through emotion regulation was significantly different from those via borderline traits alone (INE = − 0.50, BootSE = 0.22; 95% CI = − 0.97 to–0.12) and via emotion regulation alone (INE = − 0.70, BootSE = 0.28; 95% CI = − 0.25 to–0.23).

**Table 3:** Mediation estimates for the serial multiple mediation model

Effect	Estimates	BootSE	95% CI			Mediation %
Indirect (BT)	0.26	0.13	0.02–0.54			23.6
Indirect (ER)	0.06	0.13	−0.30–0.40			5.5
Indirect (BT via ER)	0.75	0.18	0.44–1.14			68.2
Total IE	1.06	0.28	0.55–1.65			96.8

*BT* Borderline traits, *ER *Emotion regulation, *IE* Indirect effect.

A follow-up analysis used the variables “Family’s economic situation during the last two years” (see Table 1) and the outcome variable depressive symptoms assessed at baseline as covariates. The results showed a nonsignificant effect on family economics, *t*(178) = 0.75, *p* = 0.45. In contrast, a significant effect was found for depressive symptoms, *t*(178) = 3.07, *p* = 0.0025. This model accounted for 61.4% of the total variance, *F*(45, 178) = 56.73, *p* < 0.0001. However, once the two mediators were controlled, the direct path from SLEs to depressive symptoms was nonsignificant (*b* = −1.04, *p* = 0.66). The indirect paths from SLEs via emotion regulation (INE = −1.04, BootSE = 0.14; 95% CI = −0.39 to 0.19) and borderline traits (INE = 0.02, BootSE = 0.06; 95% CI = −0.08 to 0.16) were also nonsignificant. Nevertheless, the specific indirect path from SLEs through borderline traits and then via emotion regulation to depressive symptoms was significant (INE = 0.13, BootSE = 0.08; 95% CI = 0.02 to 0.32). Additionally, participants reported also on traumatic events experienced over the past three years, and similar results were found. Using “reporting experience of SLEs over the last three years” as a predictor variable, the direct paths from SLEs to depressive symptoms were nonsignificant, whereas a statistically significant indirect path (INE = 0.07, BootSE = 0.05; 95% CI = 0.007 to 0.19) was identified from SLEs through borderline traits and then via emotion regulation to depressive symptoms. Using the outdated causal steps procedure proposed by Baron and Kenny [50] would likely have resulted in failing to reject the null hypothesis. This methodology is based on the incorrect assumption that a direct effect cannot be mediated if it is not statistically significant. Methodologists generally agree that the presence of a statistically significant total effect on an outcome variable is not necessary to investigate the existence of an indirect effect [30].

An additional post hoc follow-up analysis employing a third serial multiple mediation model (Process model 6) used DASS (syndromal depressive symptoms) as a dichotomous variable, taking a score of 14 or above to indicate clinical depression. This revealed a statistically significant specific IE (IND = 0.20, BootSE = 0.06, 95% CI 0.11–0.36) between SLEs and depression through borderline traits and emotion regulation. This path accounted for 55.9% of the total indirect effect. The indirect path from SLEs via borderline traits to depression was also statistically significant (IND = 0.14, BootSE = 0.07, 95% CI 0.04–0.32.). This path accounted for 39.9% of the total indirect effect. In contrast, the path from SLEs via emotion regulation to depression was not statistically significant (IND = 0.015, BootSE = 0.05, 95% CI − 0.09–0.12), and this indirect path accounted for 4.2% of the total indirect effect. Pairwise comparisons between the three indirect paths show a statistically significant difference (IND = − 0.18, BootSE = 0.08, 95% CI −0.39– − 0.07) only between the path from SLEs via borderline traits and emotion regulation compared with the path via emotion regulation to depression when the mediation variables DERS and BPFSC11 were controlled for, a reduced and statistically nonsignificant direct effect (*b* = 0.02, *p* = 0.81) was found between SLEs and depression, demonstrating full mediation. As in the former serial multiple mediation models, sex as a covariate revealed a nonsignificant difference between the sexes (*b* = 0.02, *p* = 0.98).

## Discussion

To our knowledge, this is the first reported longitudinal study using both a parallel and a serial multiple mediator model to examine the mediating effect of borderline traits and emotion regulation on the relationship between SLEs and depressive symptoms in adolescents.

As was hypothesized, we found a robust and statistically significant direct effect of SLEs on depressive symptoms. Also, when a parallel multiple mediation model was applied, the total IE as a sum of two specific IEs of SLEs via both emotion regulation and borderline traits on depressive symptoms was statistically significant, and these variables fully mediated the path from SLEs to depressive symptoms. Contrary to our hypothesis, in the serial multiple mediation model, the specific IE from SLEs via emotion regulation to depressive symptoms was not statistically significant. In contrast, the two specific indirect pathways from SLEs to depressive symptoms via borderline traits, and via borderline traits through emotion regulation were robust and statistically significant. These findings suggest that the indirect effect of SLEs on depressive symptoms, as mediated by emotion regulation, may decrease over time. Additionally, our results show that individuals with higher borderline traits are more likely to experience difficulties in emotion regulation. Moreover, a pairwise comparison across the three specific indirect paths revealed that the path from SLEs via borderline traits through emotion regulation to depressive symptoms was significantly more robust than the other two paths. This specific IE alone accounted for 70.8% of the total IE, and 68.5% of the total effect in this model.

Additionally, controlling baseline depressive symptoms at assessment point 1 allowed for a more accurate evaluation of how adverse SLEs contribute to subsequent depressive symptoms at assessment point 2. This approach reduces the risk of overestimating the impact of SLEs. As anticipated, the direct effect of SLEs on depressive symptoms at assessment point 2 became less pronounced and nonsignificant when accounting for baseline depressive symptoms due to the shared variance between the two assessment points. Baseline depressive symptoms likely diminish the apparent effect of SLEs on both emotion regulation and borderline traits. This is important because depression can influence regulatory abilities and potentially alter the strength of the mediational pathways involved. Despite this, the indirect path from SLEs to depressive symptoms, mediated by borderline traits via emotion regulation, was found to be significant. This finding provides a more nuanced and comprehensive understanding of these relationships, ensuring that the predictor (SLEs) temporally precedes the outcome (depressive symptoms) at the assessment point 2. The significance of these findings is particularly pronounced in the context of adolescent development. Adolescence is a critical window for both the emergence of borderline traits and the maturation of emotion regulation capacities. Our results suggest that adolescents who experience multiple SLEs are at increased risk for developing borderline features, which in turn exacerbate emotion regulation difficulties and elevate the risk for depressive symptoms. This developmental cascade underscores the importance of early screening and intervention targeting borderline traits and emotion regulation in adolescent mental health settings. Intervening during this formative period may not only reduce current depressive symptoms but also interrupt the trajectory toward chronic psychopathology in adulthood.

Linehan’s biosocial theory [34] suggests that individuals with BPD are genetically predisposed to emotional sensitivity, making them vulnerable to developing BPD in invalidating environments. Our results align with Linehan’s theory, indicating that borderline traits predispose individuals to emotional dysregulation. This mechanistic insight provides a compelling rationale for early, integrated interventions targeting borderline features and emotion regulation skills in adolescent populations.

These findings may have important clinical implications. A robust, significant relationship between SLEs and depressive symptoms demonstrates that paying attention to children and adolescents who experience SLEs is crucial. Examining a sample of undergraduates in the US, Frazier et al. [8] reported that 85% of the students reported having experienced at least one adverse life event during their lifetime. Aune and Stiles [51] explored a population-based sample of 11–14-year-old students, reporting that 73% had experienced at least one adverse life event during the previous year. In addition, 33% had experienced three or more negative life events in one year. The mean RFQ score of 3.18 and a standard deviation of 2.56 indicate that participants in this study reported a relatively high number of risk factors. In addition, 66% reported one to five risk factors during the previous year. Laor et al. [52] validated the cumulative effect of risk factors in adolescents exposed to continuous stress. Moreover, Baglivio [53] demonstrated that cumulative exposure to SLEs is a powerful predictor of adverse mental health, emphasizing the importance of examining the totality of stressful experiences rather than isolated adverse exposure. Nevertheless, we did not examine the impacts of different traumatic event types.

The finding that borderline traits, while simultaneously controlling for emotion regulation, have a robust and statistically specific mediational effect among adolescents has not been shown previously. This finding underlines the importance of detecting borderline traits at an early age. Although emotion regulation has unquestionably been linked to borderline traits, our results show that these two constructs share 57.2% of the common variance. The serial multiple mediation models expand the results from the parallel multiple mediation model, demonstrating that the path from SLEs via emotion regulation to depressive symptoms (accounting for only 5.3% of the total IE) is not statistically significant when we simultaneously control for the other two paths. The statistically significant IE of emotion regulation as a single mediator on the path between SLEs and depressive symptoms reported by Aune et al. [24] and Stikkelbroek et al. [25], both using a cross-sectional research design, seems to diminish when a serial multiple mediation model, including borderline traits assessed in one year before emotion regulation is used. More precisely, the association between SLEs and emotion regulation is no longer significant, indicating that this association weakens over time. Moreover, even though the specific indirect path from SLEs through borderline traits to depressive symptoms is statistically significant, the path from SLEs via borderline traits through emotion regulation to depressive symptoms is indisputably robust and significantly more substantial than the other two paths in this model. Moreover, the slopes for this path reveal a robust and significant association between SLEs and borderline traits and an even stronger relationship between borderline traits and emotion regulation. Post hoc analysis, creating scores for depression over a clinical threshold, revealed more or less the same results as the former serial mediation multiple model. The results indicate temporal precedence in therapeutic interventions, whereby modifying borderline traits prepares adolescents to be more receptive to developing suitable emotion regulation skills. Buer Christensen et al. [54] have shown that the level of personality functioning is the most important predictor of the general management of life, but also regarding symptom disorders. Moreover, borderline personality disorder has been shown to have the strongest association with impairment [55]. Karterud [56] proposes that personality functioning, like identity, integrity, setting realistic goals, ability to relate and commit, and the ability to regulate emotionally, is highlighted in both DSM-5 and ICD-11 as the most important part to assess regarding personality. Social functioning associated with depression can be partially ascribed to deficits in mentalizing [57] and the ability to regulate emotionally [58]. Rifkin-Zybutz et al. [59] showed that among depression patients, impairment in mentalization was associated with an increased level of depressive symptoms.

This study has several strengths and limitations. A longitudinal research design was used. Employing rigorous, adequate, and psychometrically sound inventories to assess emotion regulation, borderline traits, SLEs, and depressive symptoms strengthens the reliability of the results. Including a correlated mediator in the two models allowed us to disentangle spurious relationships from potential causal associations. When compared to other models [25], which include only one mediator (emotion regulation), the addition of an extra correlated mediator (borderline traits) leads to more significant sampling variance and reduced statistical power. However, the specific indirect effects in both the parallel and serial mediation models remain statistically significant. In addition, applying a path analysis on a regression frame allowed us to conduct pairwise comparisons among the two mediators and statistical estimates of whether one is significantly more robust. Although we employed a longitudinal research design with two assessment points, introducing additional assessment points would likely increase the robustness of the results; however, the assessment of SLEs at assessment point I was based on retrospective information. Participants were asked about the occurrence of SLEs during the previous year, and three assessment time points were indicated. Nevertheless, we acknowledge that measuring mediators and predictors concurrently limits our ability to establish temporal precedence conclusively. A limitation of the present study concerns the assessment of SLEs. Although checklist measures of life event exposure are widely used in research, they have well-documented psychometric limitations [12, 15]. One key issue is that such checklists do not account for the considerable heterogeneity in the subjective and objective impact of similar events across individuals. Without examining the context or perceived severity of each endorsed event, checklist approaches may fail to capture the true variability in stressor impact. Moreover, the BPFSC11 does not address all the borderline traits labeled in DSM-5 [14]. Lastly, the DERS total score was calculated from four subscales, not six. Although previous research has demonstrated high correlations among the six subscales and the presence of one general factor [60], we recognize that omitting the impulse control and emotional clarity subscales may have influenced our findings. These two domains represent essential aspects of emotion dysregulation, and their exclusion may mean that our total score does not fully reflect the multidimensional nature of the construct as initially conceptualized by Gratz and Roemer [39]. Thus, we must consider that a total score of all six subscales may yield a different result.

The results might have several implications. First, those who report a significant number of borderline features also report many SLEs in the previous year. Even though there is a temporal relationship between these two variables, Carpenter and Trull [37] demonstrated that those with BPD are more prone to accidents and traumatic experiences because of their behavior. Therefore, interventions that reduce borderline features may not only reduce the prevalence of depressive symptoms at a later stage but also lessen the probability of repeating traumatic experiences, hence avoiding vicious circles. Second, the strong and statistically significant temporal relationship between borderline traits and emotion regulation also demonstrates that working with borderline traits and emotion regulation simultaneously and over time may reduce depressive symptoms among adolescents at risk.

Thus, interventions targeting adolescents with borderline traits, in particular the regulation of emotional expression, identity difficulties, self-harm, invalidating attitudes, and relationship impairment, should be considered part of routine practice in adolescent mental health to improve their long-term well-being.

## Conclusion

A statistically significant relationship exists between experiencing stressful life events and the later development of depressive symptoms. While emotion regulation is a key feature of borderline traits, the concept of borderline features encompasses a broader range of characteristics, including various general personality disorder traits. Both borderline traits and emotion regulation mediate the link between stressful life events and depression; however, in a serial multiple mediation model, borderline traits exacerbate issues with emotion regulation. We propose replicating this analysis with longitudinal data, with rigorous tests of temporal precedence.

## Data Availability

No datasets were generated or analysed during the current study.
